# Combining No-Tillage with Hairy Vetch Return Improves Production and Nitrogen Utilization in Silage Maize

**DOI:** 10.3390/plants13152084

**Published:** 2024-07-27

**Authors:** Zhou Li, Xingrong Sun, Jie Pan, Tao Wang, Yuan Li, Xiuting Li, Shuai Hou

**Affiliations:** 1 Key Laboratory of Animal Genetics, Breeding and Reproduction in the Plateau Mountainous Region, Ministry of Education, College of Animal Science, Guizhou University, Guiyang 550025, China; zli8@gzu.edu.cn (Z.L.); likettle3891@163.com (J.P.); 2 College of Grassland Agriculture, Northwest A & F University, Yangling 712100, China; sxr15204648399@163.com (X.S.); lxt@nwafu.edu.cn (X.L.); 3 Rapeseed Research Institute, Guizhou Academy of Agricultural Sciences, Guiyang 550008, China; wangtao10062023@163.com; 4 Grasslands and Sustainable Farming, Production Systems Unit, Natural Resources Institute Finland, Halolantie 31A, FI-71750 Kuopio, Finland; yuanli@lzu.edu.cn

**Keywords:** conservation tillage, green manure, nutrient management, forage yield, economic benefits

## Abstract

The combination of no-till farming and green manure is key to nourishing the soil and increasing crop yields. However, it remains unclear how to enhance the efficiency of green manure under no-till conditions. We conducted a two-factor field trial of silage maize rotated with hairy vetch to test the effects of tillage methods and returning. Factor 1 is the type of tillage, which is divided into conventional ploughing and no-tillage; factor 2 is the different ways of returning hairy vetch as green manure, which were also compared: no return (NM), stubble return (H), mulching (HM), turnover (HR, for CT only), and live coverage (LM, for NT only). Our findings indicate that different methods of returning hairy vetch to the field will improve maize yield and quality. The best results were obtained in CT and NT in HM and LM, respectively. Specifically, HM resulted in the highest dry matter quality and yield, with improvements of 35.4% and 31.9% over NM under CT, respectively. It also demonstrated the best economic and net energy performance. However, other treatments had no significant effect on the beneficial utilization and return of nutrients. The LM improved yields under NT by boosting soil enzyme activity, promoting nitrogen transformation and accumulation, and increasing nitrogen use efficiency for better kernel development. Overall, NTLM is best at utilizing and distributing soil nutrients and increasing silage maize yield. This finding supports the eco-efficient cultivation approach in silage maize production in the region.

## 1. Introduction

Due to population growth and rising standards of living, the global per-person demand for livestock products is expected to increase by between 6 and 23 kg by 2050 [1]. This presents a significant challenge to the supply of feed [2]. In order to enhance the yield of feed, people tend to over-cultivate and fertilize, which can result in the deterioration of soil quality, the inhibition of crop growth, and an increase in production costs [3]. Consequently, it is imperative to optimize tillage measures in order to mitigate the adverse consequences.

No-tillage (NT) is a conservation tillage practice that maintains the stability of soil structure by reducing soil disturbance, thereby reducing erosion and nitrogen fertilizer loss. In turn, it improves nitrogen fertilizer efficiency [4]. It was found that compared to conventional tillage, no-tillage increased soil organic matter content by 1.12% [5], total nitrogen content by 18%, and significantly enhanced ammonium nitrogen content [6] as well as the productivity of soybean (*Glycine max* (L.) Merr.) [7]. Maize (*Zea mays* L.) also exhibited high rhizosphere soil respiration and enzyme activities under no-tillage conditions, which were attributed to the enrichment of microbial communities [8]. This phenomenon has been observed to improve the growing environment of the crop and to enhance yield by improving the agronomic traits of the plant. Concurrently, it can diminish the frequency of implements being placed into the ground and reduce the degree of compaction caused by agricultural implements on the soil. Additionally, it can mitigate the damage to the soil structure. Furthermore, it can effectively conserve energy and reduce production investment. However, Pittelkow et al. [9] found in a global meta-analysis that yields of many crops have declined when transitioning from conventional to no-till. They also found that introducing cover crops in no-till systems can mitigate these negative effects during the transition [10].

It has been demonstrated that cover crops raise soil organic matter, encourage plant development, and enhance crop quality [11]. In the right circumstances, it can be used in conjunction with no-tillage to promote plant translocation of high-C/N-ratio organic matter from above-ground biomass to below-ground biomass, enhance soil permeability, lower soil weight and temperature, encourage microbial decomposition in the rhizosphere zone, and increase soil enzyme activity [12]. The alkaline phosphatase activity was effectively increased by 13.46% to 45.03%. This increase was attributed to the return of conifer pea (*Pisum sativum* L.), sweet pea (*Pueraria montana* var.), and melilotus (*Melilotus officinalis* L.) root stubble to the field. The sweet pea pressure greening treatment had the highest urease activity with an increase of 19.75% [13]. Nevertheless, the presence of rhizosphere secretions had distinct effects on the enzyme activities of rhizosphere and non-rhizosphere soils. Specifically, the urease content in maize rhizosphere soils increased by 19.7% to 63.2%, while in non-rhizosphere soils, it increased by 28.5% to 66.5% [14]. Furthermore, the increase in enzyme activity enhanced the transformation of organic nitrogen into inorganic nitrogen, specifically, nitrate and ammonium nitrogen, which may be readily absorbed and utilized by plants [15,16]. Research has demonstrated that no-till cover crops had a 16% greater average nitrogen absorption than conventional tillage, resulting in a 12% increase in seed production [17]. Additionally, agricultural expenses were cut by 20% [18]. As of right now, using legumes as a cover crop has significantly increased the nitrogen fertility of the soil. Furthermore, the use of cover crops has led to an increase in both maize production and total N content of the soil [8]. The majority of current research [19] focuses on how various tillage techniques impact soil N transfer to plants through microorganisms. However, the mechanisms of nutrient return and regulation for different cover crops are not yet clear.

The southwestern karst mountainous regions, recognized as one of the major global locations with high concentrations of karst formations, are confronted with significant issues of soil erosion and infertility [20]. Utilizing mixtures of no-till and green manure application aids in mitigating soil erosion while facilitating soil recuperation and stabilization and enhancing crop production. Silage maize (*Zea mays* L.) is widely used for feed production owing to its high yield, energy content, and nutrient properties [21]. However, the most effective method for returning green manure to the soil and balancing nutrient maximization with maize yield preservation remains unclear. So as to assess the effectiveness of no-tillage mulching, hairy vetch-silage maize was planted in Tangtou Town, Sinan County, Tongren City, Guizhou Province. The goal was to conduct a thorough analysis and comparison of the effects of different residue return strategies and tillage techniques on the production, quality, and nitrogen utilization characteristics of maize silage. We sought to understand the underlying mechanisms of how conservation tillage in conjunction with various residue return techniques improves crop yield, nitrogen use efficiency, and soil nitrogen utilization efficiency by adjusting nitrogen distribution. We did this by looking at changes in nitrogen distribution and enzyme activity within rhizosphere and non-rhizosphere soils. The objective is to offer a theoretical foundation for the sustainable development of grassland animal husbandry in the karst mountainous regions of southwest China, as well as to identify an ecologically sound and effective cropping method appropriate for the production of maize silage in this location. 

## 2. Results

### 2.1. Components of Silage Maize Yield and Usage of Nitrogen Fertilizer

The relationship between tillage practices and techniques of returning hairy vetch residues had a substantial (*p* < 0.05) impact on plant height, leaf area index, dry matter quality, and silage maize production at the end of milk maturity. The combination of conventional and no-tillage methods, along with different return methods of hairy vetch, resulted in increased physiological indexes and yield of silage maize. The plant height, dry matter mass, and yield of CTHM and NTHM were the highest under these two measures, respectively. They were 15.08% and 12.94%, 35.40%, 27.89%, 31.89%, and 26.01% higher than that of CTNM and NTNM, respectively. Additionally, the leaf area index was significantly influenced by CTH and NTHM. The leaf area index was greatest in CTH and NTHM, with NTHM exhibiting a significant increase of 7.89% compared to NTNM. Conventional tillage procedures demonstrated significant benefits on the growth indices and productivity of silage maize (Table 1).

The combination of tillage techniques and the presence of hairy vetch had a substantial impact on the starch content, crude protein content, neutral detergent fiber, RFV (Relative Feed Value), and GI (Glycemic Index) of silage maize at the end of milk maturity (*p* < 0.05). RFV, GI, starch, and crude protein levels increased with different field return techniques. All were highest in NTLM, showing significant increases of 25.34%, 16.96%, 14.81%, and 54.13% above CTNM. The levels of crude protein and GI exhibited a significant increase of 7.74% and 17.21%, respectively, as compared to the control group (NTNM). While roughage showed a negative correlation with ADF and NDF concentrations, CTNM had the greatest levels of acidic and neutral detergent fiber. However, these levels were not significantly influenced by tillage techniques. Based on the GI grading of dairy pasture, the silage maize from the NTLM treatment was classified as a first-grade roughage with a greater feeding value (Table 2).

Interactions between tillage practices and hairy vetch return had significant effects on nitrogen harvest index, nitrogen use efficiency, and nitrogen fertilizer bias productivity (*p* < 0.001). The nitrogen harvest index, nitrogen usage efficiency, and nitrogen fertilizer bias productivity were influenced by varying tillage techniques. With a 7.65% increase over CTNM, the nitrogen harvest index was greatest in CTH and CTHM treatments under conventional; NTLM was 7.73% more than NTNM when there was no tillage. Both N use efficiency and N fertilizer bias productivity under conventional measures were highest for CTHM, 6.43% and 28.19% higher than for CTNM. In NTLM, no-tillage improved nitrogen usage efficiency by 4.83%, whereas in NTHM, nitrogen fertilizer bias productivity was greater than in NTNM by 29.33% (*p* < 0.05) (Table 3).

**Figure 1:**
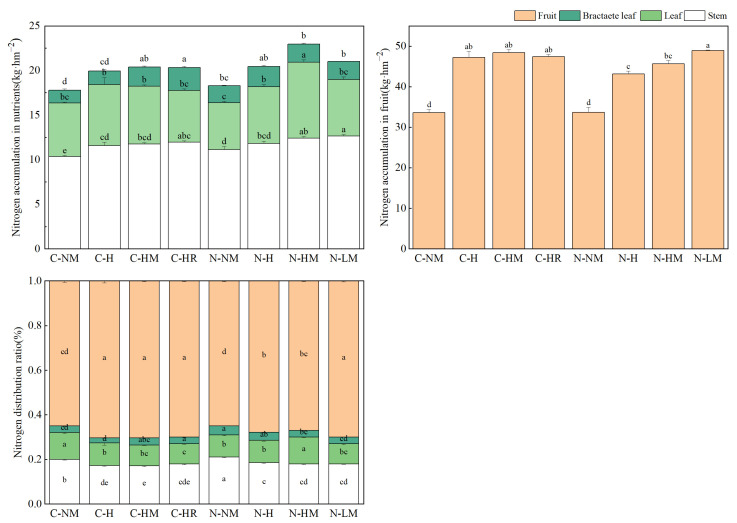
No-tillage and hairy vetch returning methods had an effect on nitrogen accumulation and nitrogen distribution ratio of various organs of silage maize at the end of milk maturity. **Note**: CTNM: conventional tillage; CTH: conventional tillage hairy vetch; CTHM: conventional tillage + hairy vetch mulch; CTHR: conventional tillage + hairy vetch pressure; NTNM: no-tillage; NTH: no-tillage + hairy vetch; NTHM: no-tillage + hairy vetch; NTLM: no-tillage + hairy vetch living mulch. Different letters indicated significant differences in nitrogen accumulation and nitrogen allocation ratio in the same organ between different treatments (*p* < 0.05).

The distribution and buildup of nitrogen varied between the silage maize organs under varying tillage and returning techniques (*p* < 0.05). Every returning treatment increased the overall amount of N accumulation; all the nitrogen allocations showed kernel > stem > leaf > bractaete leaf, with the kernel exhibiting the greatest N accumulation (average of 33 kg hm^−^^2^) and allocation (more than 60% of the entire plant amount). NTLM exhibited the highest N accumulation of kernel, with a difference of 45.79% and 45.53% compared to CTNM and NTNM (*p* < 0.05). When comparing CTH, CTHM, CTHR, and NTLM treatments to CTNM, all the nitrogen allocation tests revealed that stems contributed the most to the nitrogen transfer and allocation from silage maize bracts, leaves, and stems to kernels (Figure 1).

### 2.2. Nitrogen Content and Enzyme Activity in Rhizosphere and Non-Rhizosphere Soil

**Figure 2:**
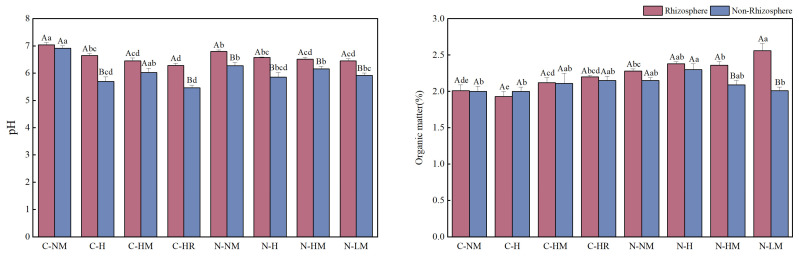
Effects of no-tillage and hairy vetch returning on pH and organic matter. **Note**: CTNM: conventional tillage; CTH: conventional tillage hairy vetch; CTHM: conventional tillage + hairy vetch mulch; CTHR: conventional tillage + hairy vetch pressure; NTNM: no-tillage; NTH: no-tillage + hairy vetch; NTHM: no-tillage + hairy vetch; NTLM: no-tillage + hairy vetch living mulch. Different lowercase letters indicate that there are significant differences in pH and organic matter content between different treatments of the same type of soil (*p* < 0.05), while different uppercase letters indicate that there are significant differences in pH and organic matter content between different types of soil under the same treatment (*p* < 0.05).

The reintroduction of hairy vetch to the field along with various tillage demonstrated a similar trend in pH impacts on rhizosphere and non-rhizosphere soils, although there were variations in the amount of organic matter present. The pH of both rhizosphere and non-rhizosphere soils was greatly lowered by all the return fields, and the rhizosphere soils had a larger organic matter content than the non-rhizosphere soils. Among these, pH dropped the greatest under CTHR in both non-rhizosphere and rhizosphere soils (11.92% and 26.51% compared to CTNM), while the highest organic matter concentration was found in NTLM rhizosphere soils (27.36% compared to CTNM) (*p* < 0.05). In contrast, NTH was the highest in the non-rhizosphere, with a 15% increase over CTNM (Figure 2).

**Figure 3:**
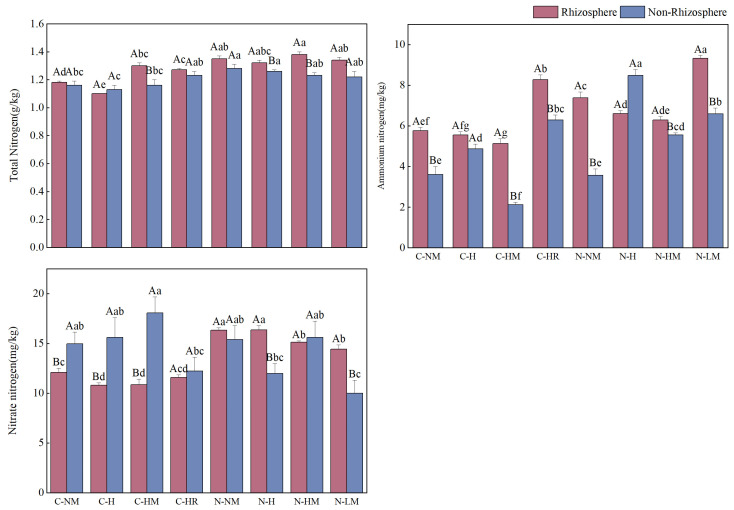
Total nitrogen, ammonium nitrogen, and nitrate nitrogen content in rhizosphere soil and non-rhizosphere soil under no-tillage and hairy vetch. **Note**: CTNM: conventional tillage; CTH: conventional tillage hairy vetch; CTHM: conventional tillage + hairy vetch mulch; CTHR: conventional tillage + hairy vetch pressure; NTNM: no-tillage; NTH: no-tillage + hairy vetch; NTHM: no-tillage + hairy vetch; NTLM: no-tillage + hairy vetch living mulch. Different lowercase letters indicate that the total nitrogen content of the same type of soil is significantly different between different treatments (*p* < 0.05), and different uppercase letters indicate that the total nitrogen content of different types of soil is significantly different between the same treatments (*p* < 0.05).

The total N and ammonium N contents of rhizosphere and non-rhizosphere soils varied depending on the hairy vetch return methods and tillage techniques used. The total and ammonium N contents were higher in no-tillage than in conventional tillage, but the nitrate N trended differently in rhizosphere and non-rhizosphere soils and was encouraged by combining different return methods. Specifically, rhizosphere total nitrogen content was highest under the NTHM treatment, with a 1.0% increase over CTNM; NTNM has 5.0% more non-rhizosphere than CTNM. Compared to the same tillage treatments of CTNM and NTNM, respectively, the rhizosphere and non-rhizosphere ammonium N content was considerably greater under CTHR and NTLM by 43.58%, 73.96%, and 26.29%, 85.11% (*p* < 0.05). However, NTH was highest in non-rhizosphere soils and was 34.9% higher than CTNM. Furthermore, all treatments—aside from total N under the CTH treatment and ammonium N content under the NTH treatment—showed greater rhizosphere than non-rhizosphere N content. While nitrate N content was higher in rhizosphere soil with NTH than in other treatments and increased by 35.4% compared to CTNM. The CTHM treatment had a nitrate N concentration that was 9.3% greater than CTNM in non-rhizosphere soils (*p* < 0.05). In non-rhizosphere soils, nitrate nitrogen levels were considerably greater than in rhizosphere soils under conventional tillage. In no-tillage, the reverse tendency was noted (Figure 3).

**Figure 4:**
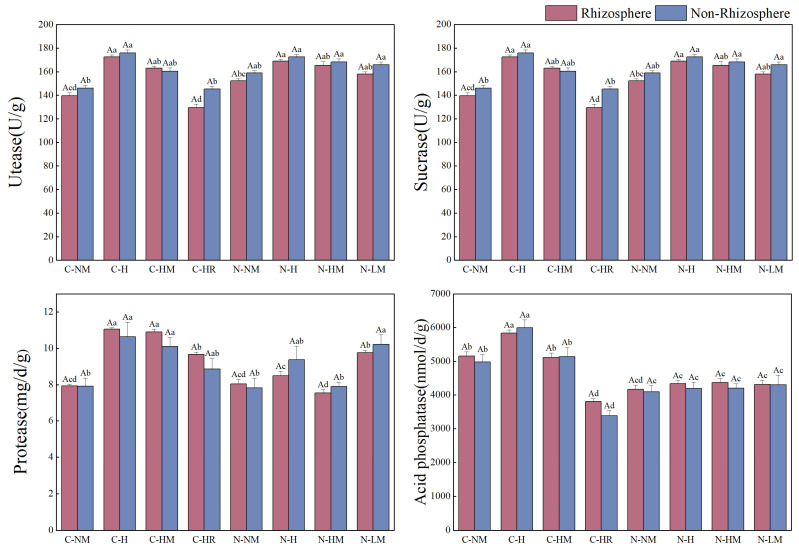
Effects of no-tillage and hairy vetch returning methods on urease activity, protease activity sucrose enzyme activity, and acid phosphatase activity in rhizosphere and non-rhizosphere soils. **Note**: CTNM: conventional tillage; CTH: conventional tillage hairy vetch; CTHM: conventional tillage + hairy vetch mulch; CTHR: conventional tillage + hairy vetch pressure; NTNM: no-tillage; NTH: no-tillage + hairy vetch; NTHM: no-tillage + hairy vetch; NTLM: no-tillage + hairy vetch living mulch. Different lowercase letters indicate that the total nitrogen content of the same type of soil is significantly different between different treatments (*p* < 0.05), and different uppercase letters indicate that the total nitrogen content of different types of soil is significantly different between the same treatments (*p* < 0.05).

Disparities in the activities of urease, sucrase, protease, and acid phosphatase in rhizosphere and non-rhizosphere soils under various plowing techniques and hairy vetch return techniques (*p* < 0.05). Urease, sucrase, and protease performance were all higher in no-tillage than in conventional tillage, while no-tillage practices had less of an impact on acid phosphatase activity, which primarily reflected changes under conventional tillage combined with different return methods; different tillage practices combined with hairy vetch return generally increased the content of each enzyme in both rhizosphere and non-rhizosphere soils. Particularly, urease increased 33.01% and 48.69%, 5.21% and 12.91% above CTNM and NTNM, respectively, in the rhizosphere and non-rhizosphere soils in the NTLM treatment. In comparison to other treatments, CTH showed considerably greater levels of sucrase, protease, and acid phosphatase, with increases of 23.47% and 20.57%, 39.47% and 34.22%, 13.22% and 20.14%, respectively, above CTNM. When no-tillage paired with return treatment, urease and sucrase rose by 10.87% and 8.6% in NTH compared to NTNM. Overall, urease and sucrase showed higher non-rhizosphere than rhizosphere, while protease and acid phosphatase showed the opposite trend (Figure 4).

The correlation analysis revealed significant differences among the indicators under no-tillage combined with various mulching measures. Plant height, dry matter content, and yield were positively correlated with the nutrient composition and nitrogen content of each component (*p* < 0.05). Notably, plant height exhibited a significant positive correlation with both dry matter content and yield. Similarly, dry matter content was significantly positively correlated with yield. The increased nitrogen content in the kernels was observed to enhance dry matter content, plant height, and yield, showing highly significant positive correlations (*p* < 0.01). Additionally, rhizosphere soil pH was found to be negatively correlated with silage maize quality and yield, indicating a significant relationship (*p* < 0.05). Soil enzyme activities, including urease, sucrase, and protease, demonstrated significant positive correlations between rhizosphere and non-rhizosphere soils. However, acid phosphatase activities showed a negative correlation with organic matter, total nitrogen, and ammonium nitrogen.

### 2.3. Economic Efficiency and Energy Balance

There were significant differences between the economic benefits of tillage practices and hairy vetch return on silage maize production systems. Compared to conventional (CTNM), no-tillage (NTNM) reduced gross inputs (7.77%) and increased gross outputs, input–output ratio (7.41%), and net income (5.08%). Under the no-tillage system, it was higher in the case of NTHM than the other treatments, which were 6.27%, 26.01%, and 36.30% higher than NTNM. In contrast, the total inputs, total outputs, and net incomes of the treatments were highest in the case of CTHM, which were 3.7%, 14.8%, and 18.7% higher than CTNM. The largest variation in total inputs across treatments was seen in labor, which constituted over 45% of the total inputs. Fertilizer inputs, on the other hand, accounted for over 27% of the total inputs. Furthermore, NTLM had the greatest production–input ratio, which was 33.33% greater than that of CTHM and 24.14% higher than that of NTNM (Table 4).

The primary energy inputs used in the production of the various treatments are labor, fuel fertilizer, and seed. In the silage maize production system, the total energy input varied between 13.91 and 18.25 (GJ·ha^−^^1^). All treatments under no-tillage input less energy than treatments under conventional tillage, with CTHM being the most abundant and 13.3% lower than CTNM. The primary source of energy output in the silage maize production system was the plant itself. However, no-tillage yielded higher results than conventional measures. When combined with the return of hairy vetch to the field, the energy output and net energy value of the system increased and were the highest in CTHM, 15.0% and 16.4% higher, respectively, than in CTNM (Table 5).

## 3. Discussion

Cover crop return tends to enhance crop productivity by enhancing the physical and chemical characteristics of the soil [22], resulting in improved economic efficiency and energy output. Our findings demonstrated that the various return methods employed in the no-tillage system had a significant impact on soil pH, soil organic matter, and total nitrogen content. Additionally, these methods facilitated the transformation of nitrogen in both rhizosphere and non-rhizosphere soils, leading to enhanced plant growth, improved plant quality, and increased utilization of nitrogen fertilizers. Furthermore, both tillage procedures resulted in variable degrees of improved maize yields when alternative return methods were employed.

### 3.1. Enhanced Yield and Nitrogen Efficiency in Different Treatments

In this experiment, maize grown with hairy vetch mulch restored to the field demonstrated the highest dry matter content and yield under various tillage techniques. Because silage maize has a higher temperature during the reproductive phase, hairy vetch mulching increases plant water utilization by decreasing water evaporation and increasing plant-accessible water and leaf area index [23] and, thus, promotes the growth of maize.

Greater efficiency in the use of light resources by taller plants results in an increase in photosynthetic area [24], improved energy conversion efficiency, increased accumulation of starch and other components, and an improvement in the production and quality of maize. Under CTHM, nitrogen utilization efficiency and nitrogen harvest index can be improved by reducing nitrogen loss in runoff and infiltration [25]. The nitrogen fixation of the legume hairy vetch, which can effectively take up mineral N from the soil and show significant effects in fixing N as well as improving soil N fertility [8], promoted nitrogen transfer to the kernel, meant that NTLM nitrogen accumulation and utilization efficiency was the highest when compared to CTHM, despite no-tillage reducing N losses.

In addition, NTHM may have reduced surface soil moisture infiltration, affecting crop water availability, and reduced surface soil moisture can limit maize growth [26], especially under drought conditions. The presence of a cover crop may temporarily reduce soil aeration, affecting root respiration, and consequently cause maize yields to suffer under NTHM, the accumulation of organic matter in the soil surface layer under NTHM treatments may cause maize roots to be more densely distributed in the surface soil layer, which may affect root uptake of water and nutrients from the deeper soil layers [27].

Higher maize yields than NTH were achieved by increasing the nitrogen harvest index and bias productivity, as well as by enhancing soil aeration and water infiltration under CTH and burying root stubble into the soil, which increased sucrase and acid phosphatase activities in the soil and facilitated root uptake of more nutrients from the soil [28]. Furthermore, CTH showed a greater leaf area index, which improved ultimate production by increasing kernel nitrogen accumulation. 

It is important to mention that while the yield of CTHR was 3.47% higher than that of NTLM, it stores a significant amount of nitrogen in its underground portion after the aboveground part of the hairy vetch is mowed. This allows nitrogen-converting microorganisms in the soil to release fixed nitrogen again, satisfying the increased nitrogen demand of maize and demonstrating a greater accumulation and utilization of nitrogen [8]. Additionally, it fosters a more advantageous environment by controlling soil pH and temperature and accumulating soil organic matter. This, in turn, boosts the stability of soil aggregates and improves the efficiency of nutrient use [29]. Salmerón et al. [30] showed that tilling just green manure can result in inadequate nitrogen mineralization in the soil, or nitrogen mineralization may not align with maize intake. To get optimal results, a certain amount of fertilizer must be applied. Furthermore, it was discovered that the use of CTHR and NTLM treatments facilitated the transfer of nitrogen from silage maize bracts, leaves, and stems to kernels, with stems being the primary contributor.

The elevated levels of starch and crude protein in NTLM serve as a source of energy and amino acids for the plant. This boosts the growth of roots, enhances nitrogen absorption, and facilitates the overall development of maize [31,32]. Consequently, these factors contribute to the improvement of RFV (Relative Feed Value) and GI (Growth Index) values.

### 3.2. Enhanced Enzyme Activity Facilitates the Conversion and Buildup of Nitrogen in Both Rhizosphere and Non-Rhizosphere Soils

Soil organic matter serves as a crucial indication for evaluating the quality of soil [33]. Conservation tillage enhances soil structure and soil ecology by minimizing disruption to agricultural soils. Additionally, increasing the organic matter content of soils promotes the retention of organic nitrogen in soils. This study discovered that the combination of tillage practices and various methods of returning hairy vetch to the soil resulted in a decrease in pH levels in both rhizosphere and non-rhizosphere soils; this can be attributed to the fact that the return of hairy vetch to the field stimulated the activity of soil microbial communities and expedited the decomposition of crop residues in the topsoil. As a result, organic acids were released into the soil, leading to a reduction in soil pH [34] and accelerating the process of mineralization of organic nitrogen and the release of more ammonium nitrogen. At the same time, both rhizosphere and non-rhizosphere soils’ organic matter content increased, which contributed positively to the accumulation of soil N pools. The NTLM treatment exhibited the highest organic matter content in the rhizosphere soil, thanks to the robustness of the living mulch root system. This system promotes the growth of beneficial microorganisms and enhances organic matter through root secretions [35].

Furthermore, the use of various return methods in no-tillage farming leads to reduced soil disturbance, which, in turn, enhances fungal growth efficiency and the ratio of fungal–bacterial activity. Additionally, it decreases the processes of organic nitrogen mineralization and decomposition, as well as the release of both inorganic and organic nitrogen from decomposing crop roots and straw [36]. As a result of these factors, the overall soil nitrogen content is increased, with the highest levels observed in the rhizosphere areas under the no-tillage with high-mulching treatment (NTHM).

Nevertheless, when considering the root stubble return, both measurements exhibited a declining pattern in soil total nitrogen. The return of root stubble to the soil can lead to an increase in microbial activity, particularly those associated with nitrogen transformation. The actions of these microbes can facilitate the conversion of organic nitrogen into minerals in the soil, while also potentially increasing the processes of denitrification; whereas denitrification can lead to the emission of nitrogen into the atmosphere in the form of nitrogen gas or nitrogen oxides [37]. This reduces the total nitrogen content of the soil. 

The study also discovered that the impact of various tillage methods on the ammonium nitrogen levels in soils between rhizosphere and non-rhizosphere soils followed a similar pattern. The use of no-till with living mulch (NTLM) was found to enhance urease activity, leading to increased formation of ammonium nitrogen and, consequently, improved the effectiveness of nitrogen fertilizer utilization. Nevertheless, the impact of nitrate nitrogen on rhizosphere and non-rhizosphere areas exhibited contrasting patterns.

**Figure 5:**
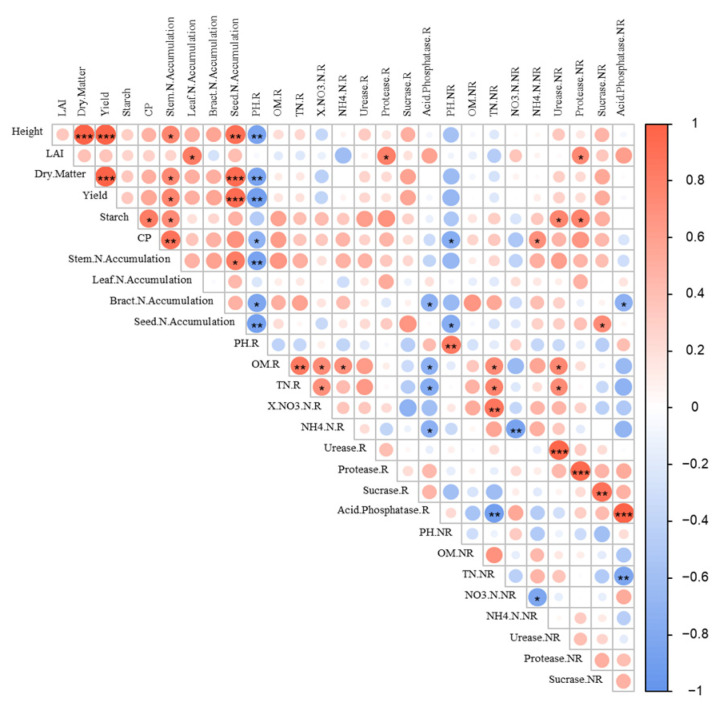
Correlation analysis of yield, quality, nitrogen use efficiency of organs, nitrogen accumulation, nitrogen distribution ratio, soil enzyme activity of silage maize. ***, **, and * indicate that the differences between treatments are 0.001, 0.01, and 0.05.

Specifically, the nitrate nitrogen content in non-rhizosphere soils was significantly higher under conventional tillage compared to no-tillage, where the conversion of ammonium nitrogen to nitrate nitrogen under CTHM was lowest in non-rhizosphere soils and high in nitrate nitrogen. This is because the disturbance caused by ploughing destroys the original soil structure, improving aeration in the arable layer. As a result, fertilizers can better dissolve into the soil, enhancing protease activity and promoting nitrification. This leads to a higher accumulation of nitrate nitrogen in the soil [38]. The nitrate nitrogen concentration is greater in no-till rhizosphere soils due to the avoidance of disturbance. However, conventional tillage also leads to nitrate nitrogen leaching when rhizosphere soils experience runoff losses following precipitation [39]. Furthermore, when subjected to CTH and CTHM treatments, the levels of urease and protease activities in the soil were elevated. However, there was no corresponding rise in the content of ammonium nitrogen and nitrate nitrogen. This suggests that the enhanced absorption of nitrogen by plants led to an increase in the nitrogen harvest index and biased productivity. Conversely, in the context of no-tillage, particularly in the NTH treatment, heightened enzyme activity facilitated the conversion of nitrogen. Yet this led to reduced nitrogen harvest index and nitrogen productivity, resulting in elevated levels of nitrate and ammonium nitrogen in the soil. The significance of soil chemical properties for crop growth was further demonstrated by correlation analyses of soil enzyme activities with organic matter and nitrogen content (Figure 5). These analyses included a negative correlation between phosphatase activities and organic matter, total nitrogen, and ammonium nitrogen in rhizosphere soils, as well as a negative correlation between rhizosphere soil pH and silage maize yield and quality. 

### 3.3. Comparison of Economic Benefits and Energy Balance

Given its close relationship to both crop yields and production costs, economic efficiency is the most logical measure of efficiency increases in maize production. Cropping patterns are mostly determined by the number of inputs and money that farmers receive. Systems of no-till cover crops increase production using fewer resources, cut down on wasteful energy consumption, and boost economic efficiency [40]. According to our study, no-tillage decreased overall energy inputs by 30.34% and economic benefits by 7.77% when compared to conventional tillage. This was explained by the fact that no-tillage required less labor and mechanical fuel. Due to increased human labor, both tillage techniques increased economic and energy inputs after cover crop return. Nevertheless, generally, these inputs were lower than those of the uncovered treatment, indicating that the cover crop return mode is more appealing in terms of financial gains [40].

The addition of crop cover through conventional tillage and no-tillage methods resulted in an increase in gross energy, economic output, output/input ratio, and net income. However, the CTHM treatment showed the highest levels of these effects because the biomass of the cover crop was tilled into the soil and its energy was counteracted, primarily by the production of silage maize. These findings are consistent with earlier research [41]. It was also demonstrated that various tillage techniques in conjunction with mulching could enhance crop production systems’ energy efficiency and boost economic viability. 

## 4. Materials and Methods

### 4.1. Experimental Site

**Figure 6:**
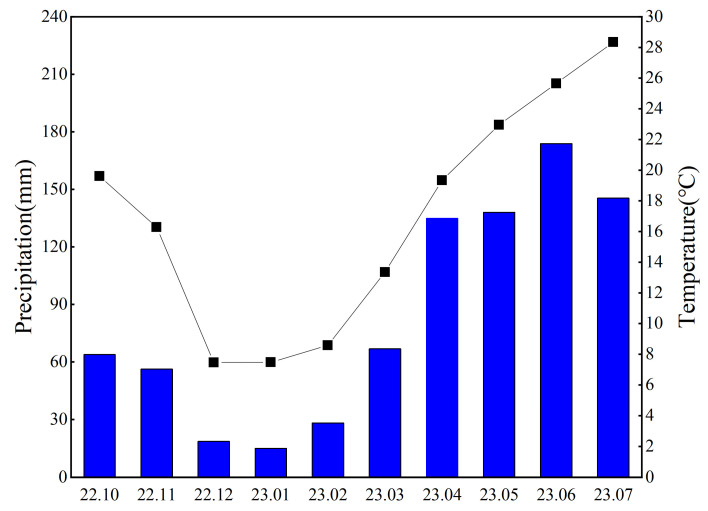
Average monthly temperature and monthly rainfall during the crop growth period October 2022–July 2023.

The Tangtou Oilseed Rape Research Institute, located in Sinan County, Guizhou Province, China (27°44′ N, 108°11′ E, 386 m), was the site of the field tests. The region is characterized by a mid-subtropical monsoon humid climate. There are 1100 h of useful sunlight and 1300 mm of precipitation in the region annually. The rainfall and average temperature during the growing period were 840.6 mm and 16.91 °C, respectively (Figure 6). There are 294 days during which there is no frost. According to the WRB (World Reference Base for Soil Resources), 4th edition, 2022, the test site soil type is categorized as red soil, N 11.25 kg/ha P 15.174 kg/ha K 21.285 kg/ha, and specific characteristics are presented in Table 6.

### 4.2. Experimental Design and Field Management

The overall experiment was divided into two parts; we conducted a two-factor field trial of silage maize rotated with hairy vetch (*Vicia villosa* Roth), the hairy vetch was sown on 22 October 2022 at a row spacing of 20 cm and an inter-row spacing of 20 cm. The sowing rate was 60 kg per hectare. The base fertilizer comprised a compound fertilizer (N 15%, P 15%, and K 15%), phosphorus pentoxide (P_2_O_5_ 12%), and potassium sulfate (K_2_SO_4_ 60%), applied at a rate of 5 kg·ha^−1^. 

On 14 April 2023, the cover crop hairy vetch was harvested, and we conducted a split-plot design with two treatments: conventional tillage (CT) and no-tillage (NT) for cultivating hairy vetch. Each treatment was replicated nine times, with six plots left fallow, totaling 24 plots. Five kinds of hairy vetch returning methods were implemented: no returning (NM), stubble returning (H), mulching (HM), turnover (HR, only tillage treatment), and live coverage (LM, only no-tillage treatment). This design yielded eight distinct treatments, each replicated three times and arranged in a randomized block design with 24 plots, each measuring 32.4 m^2^ (6 m × 5.4 m) (Table 7). On this basis, maize was grown in these eight treatments. The variety selected was HeYu 36, with a planting density of 81,000 plants per hectare, a plant spacing of 20 cm, and alternating row spacings of 80 cm (wide row) and 40 cm (narrow row). Prior to sowing, a compound fertilizer was applied at a rate of 150 kg per hectare (N 15%, P_2_O_5_ 15%, K_2_O 15%). An additional top-dressing of the same compound fertilizer was applied at the jointing stage, and urea was applied at the tasseling stage at a rate of 300 kg per hectare (N 46.7%). During the growth period of the silage maize, manual weeding was employed, and other management practices were conducted according to local agricultural practices. During the experimental period, conventional tillage (CT) involved pre-sowing cultivation to a depth of 30 cm using a rotovator, while no-tillage (NT) was maintained throughout the experimental period without any soil disturbance.

### 4.3. Measurements, Calculations and Methodologies

#### 4.3.1. Meteorological Data

Meteorological data were collected from an automatic meteorological station (YGGL-QXZ, Beijing Cloud Valley Silicon Technology Co., Ltd., Beijing, China), located 20 m from the experimental site, which meticulously recorded meteorological data such as temperature and rainfall throughout the experimental period.

#### 4.3.2. Soil Basic Physical and Chemical Properties

Following the harvest of hairy vetch (prior to the sowing of silage maize), stratified sampling was conducted at soil depths of 0–5, 5–10, 10–20, 20–30, 30–45, 45–60, 60–80, and 80–100 cm. Each stratum of soil was thoroughly mixed, and after the removal of sand, stones, and remnants of plants and animals, the soil samples were air-dried naturally. Subsequently, the soil was ground and sieved through a 2 mm (10 mesh) sieve for the determination of routine nutrients.

#### 4.3.3. Determination of Yield and Quality of Silage Maize

On 29 July 2023, at the end of the milky ripening stage of silage maize, three representative plants with uniform growth were selected from each plot, harvested at ground level, and their fresh weight, plant height, leaf length, and leaf width were measured. The number of ears, the number of kernels per ear, and the weight of 100 kernels were counted to calculate the grain yield. The leaves, stem, leaf, and fruit of each organ were separately placed in an oven at 105 °C for 30 min to inactivate the enzymes, then dried at 65 °C to a constant weight, and the dry matter weight of each organ was weighed. After drying, each organ sample was ground using a sample grinder and sieved through 0.71 mm (24 mesh) and 0.42 mm (40 mesh) sieves. 

The contents of acid detergent fiber (ADF) and neutral detergent fiber (NDF) were determined using the Van Soest method with sodium dodecyl sulfate, sodium hydroxide, and sulfuric acid. The Kjeldahl method was used to determine the nitrogen content with reagents such as sulfuric acid, copper sulfate, sodium hydroxide, and others, and then the nitrogen content was multiplied by a conversion factor of 6.25 to calculate the crude protein (CP) content. The starch content was determined by enzymolysis method with α-amylase and iodine solution [42]. The nitrogen content in various organs of silage maize is determined using the Kjeldahl method, utilizing sulfuric acid, copper sulfate, and potassium sulfate reagents [43]. 

#### 4.3.4. Leaf Area Index (LAI)

Leaf area index (LAI) is the total one-sided area of leaf tissue per unit ground surface area.

(1) $$S_{1}=L\times W\times 0.75$$

(2) $$LAI=\frac{S_{1}\times n}{A}$$

In the formula, S_1_ represents the leaf area of a single corn plant (cm^2^), L denotes the leaf length (cm), W signifies the leaf width (cm), the leaf area coefficient is 0.75 (for unexpanded leaves), the leaf area coefficient is 0.5, n is the number of maize plants per unit area, and A is the area of land in square meters (m^2^).

#### 4.3.5. Relative Feed Value (RFV)

The Relative Feed Value (RFV) is defined by comparing the voluntary intake of digestible dry matter (DDM) of a feed to that of a specific standard forage.

(3) $$RFV=\frac{DM1\times DDM}{1.29}$$

(4) $$DM1=\frac{120}{NDF}$$

(5) DDM=88.96−0.779×ADF

In the formula, DM1 represents the dry matter intake as a percentage of body weight, DDM stands for the digestible dry matter content as a percentage, NDF denotes the neutral detergent fiber as a percentage, and ADF signifies the acid detergent fiber as a percentage.

#### 4.3.6. Grading Index (GI)

The Grading Index (GI) is an indicator used for assessing the nutritional quality of roughage feed.

(6) $$VDMI=1.2\times \frac{BW}{NDF}$$

(7) $${NE}_{L}\left(MJ/kg\right)=\left[1.085-\left(0.0124\times ADF\right)\right]\times 9.29$$

(8) $$GI={NE}_{L}\times VDMI\times \frac{CP}{NDF}$$

In the formula, VDMI denotes Voluntary Dry Matter Intake of forage, BW refers to Body Weight, NDF denotes the neutral detergent fiber as a percentage, ADF signifies the acid detergent fiber as a percentage, NE_L_ represents Net Energy for Lactation, CP stands for Crude Protein, and with a calculation assumption of 600 kg for the cow.

#### 4.3.7. Nitrogen Distribution and Utilization Efficiency in Maize Organs

Understanding how nitrogen is utilized and distributed among the various organs of maize is crucial for elucidating the nitrogen metabolic mechanisms within plants. Specific calculation methods are as follows:

The nitrogen accumulation in each organ (kg·hm^−2^) = Dry matter weight of each organ × Nitrogen content of each organ (9)

Total plant nitrogen content (g·kg^−1^) = Total nitrogen accumulation in all organs/Total dry matter weight of the whole plant (10)

Nitrogen distribution rate in each organ (%) = Nitrogen accumulation in each organ/Total plant nitrogen content(11)

Nitrogen harvest index (%) = Nitrogen accumulation in the seeds/Total nitrogen absorption by the plant × 100 (12)

Nitrogen utilization efficiency (%) = Seed yield/Total plant nitrogen content × 100 (13)

Nitrogen fertilizer partial productivity (kg·kg^−1^) = Seed yield/Applied nitrogen amount (14)

#### 4.3.8. Nitrogen and Enzyme Activity in Rhizosphere vs. Non-Rhizosphere Soils

During the late lactation phase of silage maize, following the harvest of the aerial parts and the clearance of stones and detritus from the soil surface surrounding the plants, soil profiles are excavated in a quadrilateral pattern (each measuring 20 cm in length, 20 cm in width, and 40 cm in height) to isolate the entire soil core along with its root system. Both the rhizosphere and non-rhizosphere soils, once collected, are homogenized and transported to the laboratory for desiccation. Post-desiccation, both types of soil are pulverized and passed through 1 mm (18 mesh) and 0.15 mm (100 mesh) sieves. The extraction is conducted using a water-to-soil ratio of 5:1, and pH is ascertained employing a Mettler-Toledo SevenCompact S210 pH meter. The organic matter content is quantified via the external heating-potassium dichromate volumetric method [44]. Ammonium nitrogen is determined using the KCl extraction–indophenol blue colorimetric method followed by spectrophotometry [45]. Nitrate nitrogen is determined using the KCl extraction-colorimetric method. Urease activity is measured using the phenol–sodium hypochlorite colorimetric method. Protease activity is assessed using the ninhydrin colorimetric method. Acid phosphatase activity is determined using the disodium phenyl phosphate colorimetric method. Sucrase activity is measured using the 3,5-dinitrosalicylic acid colorimetric method.

#### 4.3.9. Economic Benefits and Energy Balance

In the production of silage maize, production inputs and post-harvest outputs fluctuate between treatments and can be used to gauge how economically sustainable a given treatment is. The trial real agricultural inputs, from silage maize planting to harvesting, were meticulously documented and utilized to determine the trial average cost of production. Human labor and machinery were estimated using local real-time hire and rental pricing, whereas farm inputs were based on actual market purchase prices (1 RMB ≈ $0.1376) (Table 8). The depreciation cost of the machinery was overlooked because it was rented locally and utilized in the manufacturing system (rotary tiller).

Energy inputs, both direct and indirect, are used in the production of silage maize. Manpower and diesel fuel are examples of direct energy inputs. Seed and fertilizer are examples of indirect energy inputs. The energy output was calculated by multiplying the kernel and stover of silage maize under various treatments by the corresponding conversion coefficients, as per the findings [46,47] and others, which discovered that energy input can be obtained by multiplying direct and indirect energy inputs (Table 9) by their corresponding energy conversion coefficients (Table 10).

(15) $$E_{input}=\sum_{i=1}^{n}N_{i}$$

(16) $$E_{output}=SP\times EF$$

(17) $${Net}_{E}=E_{input}-E_{output}$$

In the formula, the total energy input is represented by *Einput*, the total energy output by *Eoutput*, the energy consumption of each item (MJ·ha), the number of agro-materials input items is represented by *I*, the harvested seeds and straw are represented by *SP*, and the corresponding energy factor conversion factor is represented by *EF*, and the net energy is represented by *NetE*. 

### 4.4. Statistical Analysis

The experimental data were recorded and preliminarily organized using Microsoft Excel 2019. Statistical analysis of the collated data was performed using SPSS 20.0 software. The results were presented as mean values with standard errors. Two-way ANOVA was conducted to assess the effects of different cover crop utilization methods within the same tillage treatments and vice versa. Tests for homogeneity of variance and normality were applied to all data sets. Duncan’s test was used for post hoc multiple comparison and significance analysis. Pearson’s method was employed for correlation analysis. Graphical and tabular representations were generated using Excel 2019, R-4.2.1, and Origin 2022.

## 5. Conclusions

No-till and hairy vetch returning has significant advantages in enhancing maize yields, quality, and nitrogen utilization. Our study reveals that through the augmentation of diverse enzyme activities in both rhizosphere and non-rhizosphere soils, they enabled the conversion of nitrogen and more effective distribution and use, culminating in higher crop yields and enhanced energy efficiency, which in turn brought about superior economic advantages. In comparison to conventional tillage, LM demonstrated superior performance under NT, greatly aided in the transfer of nitrogen from the stem to the kernel, increasing the crop’s nitrogen bias productivity, yield, plant height, dry matter accumulation, net income, and net energy value. Additionally, it exhibited higher rhizosphere and non-rhizosphere enzyme activities, leading to an increase in available nitrogen content in the soil. In conclusion, on the basis of the yield and nutrient distribution and utilization consideration, the joint application of live mulch and no-tillage is highly suitable for the research area. The results of this study offer valuable insights into a fresh approach to promoting sustainable agricultural growth.

## Figures and Tables

**Table 1:** Effects of no-tillage and hairy vetch returning methods on morphological index and yield of silage maize.

Treatment	Height (cm)	Leaf Area Index	Yield (t·ha^−1^)	Total Dry Matter (t·ha^−1^)
CTNM	259.6 c	0.39 abc	46.8542 e	13.5664 d
CTH	278.5 abc	0.41 a	55.8613 cd	16.5421 b
CTHM	298.8 a	0.40 abc	61.7967 a	18.3692 a
CTHR	290.5 ab	0.38 c	59.7937 ab	17.3431 b
NTNM	258.8 c	0.38 bc	47.0977 e	13.6247 d
NTH	271.9 bc	0.40 ab	52.9918 d	15.1701 c
NTHM	292.2 ab	0.41 a	59.3490 ab	17.4251 b
NTLM	287.9 ab	0.39 abc	57.7864 bc	16.9139 b

**Note**: CTNM: conventional tillage; CTH: conventional tillage + hairy vetch; CTHM: conventional tillage + hairy vetch mulch; CTHR: conventional tillage + hairy vetch pressure; NTNM: no-tillage; NTH: no-tillage + hairy vetch; NTHM: no-tillage + hairy vetch; NTLM: no-tillage + hairy vetch living mulch. Different lowercase letters in the same column indicate significant differences between different treatments of the same index (*p* < 0.05).

**Table 2:** Effects of no-tillage and hairy vetch returning methods on silage maize quality.

Treatment	Starch (%)	Crude Protein (%)	Acid Detergent Fiber (%)	Neutral Detergent Fiber (%)	RFV	GI
CTNM	29.87 c	6.9 e	29.27 a	54.5 a	113.06 c	11.27 e
CTH	35.71 ab	7.82 ab	29.08 a	51.58 ab	119.61 bc	14.26 cd
CTHM	34.69 ab	7.45 d	26.26 a	53.67 a	118.88 bc	13.19 de
CTHR	32.81 bc	7.75 bc	28.66 a	54.2 a	114.39 c	12.89 de
NTNM	35.56 ab	7.49 cd	26.50 a	50.64 bc	125.6 ab	14.82 bcd
NTH	35.76 ab	7.96 ab	27.99 a	50.52 bc	123.74 ab	15.42 abc
NTHM	35.09 ab	7.9 ab	29.42 a	48.23 cd	127.5 ab	16.43 ab
NTLM	37.44 a	8.07 a	29.55 a	47.28 d	129.8 a	17.37 a

**Note:** CTNM: conventional tillage; CTH: conventional tillage hairy vetch; CTHM: conventional tillage + hairy vetch mulch; CTHR: conventional tillage + hairy vetch pressure; NTNM: no-tillage; NTH: no-tillage + hairy vetch; NTHM: no-tillage + hairy vetch; NTLM: no-tillage + hairy vetch living mulch. Different lowercase letters in the same column indicate that the same quality index has significant differences in different treatments (*p* < 0.05).

**Table 3:** Effects of no-tillage and hairy vetch returning on nitrogen utilization.

Treatment	Nitrogen Harvest Index (%)	Nitrogen Use Efficiency (%)	Partial Productivity of Nitrogen Fertilizer (kg·kg^−1^)
CTNM	65.33 cd	46.50 c	14.51 c
CTH	70.33 a	48.19 b	17.69 a
CTHM	70.33 a	49.49 b	18.60 a
CTHR	70.00 a	48.34 b	17.89 a
NTNM	64.67 d	49.27 b	13.98 c
NTH	68.00 b	45.94 c	15.96 b
NTHM	66.33 c	48.23 b	18.08 a
NTLM	69.67 a	51.65 a	17.78 a

**Note**: Different lowercase letters in the same column indicate that the same quality index has significant differences in different treatments (*p* < 0.05).

**Table 4:** Total input, total output, output–input ratio, and net income of silage maize production system under different treatments ($·ha^−^^1^).

Treatment	Seed	Fertilizer	Labor Hour	Diesel	Total Input	Total Output	Output–Input Ratio	Net Income
CTNM	206.4	317.86	474.72	77.61	1076.58	2901.21	2.7	1824.63
CTH	206.4	317.86	495.36	77.61	1097.22	3458.96	3.2	2361.74
CTHM	206.4	317.86	557.97	77.61	1159.83	3826.45	3.3	2666.62
CTHR	206.4	317.86	537.33	77.61	1139.19	3702.43	3.3	2563.24
NTNM	206.4	317.86	474.72	0	998.98	2916.28	2.9	1917.30
NTH	206.4	317.86	495.36	0	1019.62	3281.25	3.2	2261.63
NTHM	206.4	317.86	537.33	0	1061.58	3674.90	3.5	2613.32
NTLM	206.4	317.86	458.21	0	982.46	3578.12	3.6	2595.66

**Note**: CTNM: conventional tillage; CTH: conventional tillage hairy vetch; CTHM: conventional tillage + hairy vetch mulch; CTHR: conventional tillage + hairy vetch pressure; NTNM: no-tillage; NTH: no-tillage + hairy vetch; NTHM: no-tillage + hairy vetch; NTLM: no-tillage + hairy vetch living mulch.

**Table 5:** Energy input and output in silage maize production system under different treatments (GJ·ha^−^^1^).

Treatment	Input							Output			
	Seed	Fuel	N	P_2_O_5_	K_2_O	Labor Hour	Total Input	Maize Straw	Maize Yield	Total Output	Net Energy Value
CTNM	0.59	4.22	11.09	1.05	0.50	0.67	18.13	93.98	90.65	184.63	166.49
CTH	0.59	4.22	11.09	1.05	0.50	0.70	18.16	114.59	110.53	225.12	206.96
CTHM	0.59	4.22	11.09	1.05	0.50	0.79	18.25	127.25	122.74	249.99	231.74
CTHR	0.59	4.22	11.09	1.05	0.50	0.76	18.22	120.14	115.88	236.02	217.80
NTNM	0.59	0	11.09	1.05	0.50	0.67	13.91	94.38	91.04	185.42	171.51
NTH	0.59	0	11.09	1.05	0.50	0.70	13.94	105.09	101.36	206.45	192.51
NTHM	0.59	0	11.09	1.05	0.50	0.76	14.00	120.71	116.43	237.14	223.14
NTLM	0.59	0	11.09	1.05	0.50	0.65	13.88	117.17	113.02	230.18	216.30

**Note**: CTNM: conventional tillage; CTH: conventional tillage hairy vetch; CTHM: conventional tillage + hairy vetch mulch; CTHR: conventional tillage + hairy vetch pressure; NTNM: no-tillage; NTH: no-tillage + hairy vetch; NTHM: no-tillage + hairy vetch; NTLM: no-tillage + hairy vetch living mulch.

**Table 6:** Soil physicochemical properties of the study site.

Soil Layer	pH	Soil Organic Carbon	Total Nitrogen	Nitrate Nitrogen	Ammonium Nitrogen	Total Phosphorus	Available Phosphorus	Total Potassium	Rapidly Available Potassium
(cm)		(g·kg^−1^)	(g·kg^−1^)	(mg·kg^−1^)	(mg·kg^−1^)	(g·kg^−1^)	(mg·kg^−1^)	(g·kg^−1^)	(mg·kg^−1^)
0–5	5.29	20.71	1.74	52.00	2.82	0.74	35.64	22.79	247.97
5–10	5.39	20.52	1.52	36.75	1.23	0.66	30.92	22.87	104.91
10–20	5.37	18.39	1.49	23.60	0.50	0.67	35.17	22.24	84.61
20–30	5.73	16.1	1.17	12.01	0.30	0.61	25.08	22.49	73.30
30–45	5.93	14.73	1.07	9.73	0.26	0.53	18.65	23.27	70.25
45–60	5.91	13.76	0.94	9.31	0.25	0.62	18.28	23.63	76.59
60–80	5.80	15.24	1.13	7.49	0.91	0.48	19.7	23.60	85.28
80–100	5.68	16.03	1.21	11.78	1.09	0.48	19.41	23.85	84.86

**Table 7:** Experimental treatment and abbreviation.

Plant Date	Treatment	Abbreviation
October 2022	Conventional tillage with fallow	CT (Conventional tillage)
	No-tillage with fallow	NT (No-tillage)
April 2023	Conventional tillage with fallow (Control)	CTNM (Conventional tillage)
	Conventional tillage with stubble incorporation (Aboveground biomass removed)	CTH (Conventional tillage hairy vetch)
	Conventional tillage with mulch cover (Aboveground biomass mowed at ground level)	CTHM (Conventional tillage hairy vetch mulch)
	Conventional tillage with incorporation of aboveground biomass (Mowed and incorporated)	CTHR (Conventional tillage hairy vetch pressure)
	no-till fallow	NTNM (No-tillage)
	No-till with stubble incorporation (Aboveground biomass removed)	NTH (No-tillage vetch)
	No-till with mulch cover (Aboveground biomass mowed at ground level)	NTHM (No-tillage hairy vetch mulch)
	No-till with living mulch (Stubble left at 5 cm height)	NTLM (No-tillage vetch living mulch)

**Table 8:** Input cost of agricultural materials.

Item	Unit Price ($)	Unit
Maize seeds	5.51	kg
Silage maize stalk	0.06	kg
Compound fertilizer	0.74	kg
Urea	0.58	kg
Diesel	1.03	L
Labor	1.38	h

**Table 9:** Input of agricultural materials under different treatments in silage maize production system.

Treatment	Seed (kg·ha)	Compound Fertilizer (kg·ha)	Urea (kg·ha)	Labor (kg·ha)	Diesel (L·ha)
CTNM	37.5	300	300	345	75
CTH	37.5	300	300	360	75
CTHM	37.5	300	300	405.5	75
CTHR	37.5	300	300	390.5	75
NTNM	37.5	300	300	345	0
NTH	37.5	300	300	360	0
NTHM	37.5	300	300	390.5	0
NTLM	37.5	300	300	333	0

**Note**: CTNM: conventional tillage; CTH: conventional tillage + hairy vetch; CTHM: conventional tillage + hairy vetch mulch; CTHR: conventional tillage + hairy vetch pressure; NTNM: no-tillage; NTH: no-tillage + hairy vetch; NTHM: no-tillage + hairy vetch; NTLM: no-tillage + hairy vetch living mulch.

**Table 10:** Energy conversion coefficient of different treatments in silage maize production system.

Item	Unit	Energy Equivalent (MJ·Unit^−1^)
Inputs		
Diesel fuel	L	56.31
Seeds	kg	15.7
Nitrogen(a)N	kg	60.6
Phosphate(b)P_2_O_5_	kg	23.44
Potassium(c)K_2_O	kg	11.15
Labor hour	h	1.95
Output		
Yield	kg	14.7
Straw	kg	12.7

## Data Availability

The data presented in this study are available on request from the corresponding author.
